# Correction to: Premature Infants Display Discriminable Behavioral, Physiological, and Brain Responses to Noxious and Nonnoxious Stimuli

**DOI:** 10.1093/cercor/bhac138

**Published:** 2022-03-21

Premature Infants Display Discriminable Behavioral, Physiological, and Brain Responses to Noxious and Nonnoxious Stimuli

Marianne van der Vaart, Caroline Hartley, Luke Baxter, Gabriela Schmidt Mellado, Foteini Andritsou, Maria M Cobo, Ria Evans Fry, Eleri Adams, Sean Fitzgibbon, Rebeccah Slater

*Cerebral Cortex*, bhab449, doi: https://doi.org/10.1093/cercor/bhab449

The revised article entitled “Premature Infants Display Discriminable Behavioral, Physiological, and Brain Responses to Noxious and Nonnoxious Stimuli” by van der Vaart, et al. has been submitted by the authors as a replacement for the originally published version of the same article. The original uncorrected proof was mistakenly published due to an error by the Publisher. The corrected version of the article that has now published contains the following changes detailed below.

- Updated Supplementary Material
- Replacement of figures 3, 4, 5, 6, 7
- The authors noticed that the age of one infant included in the study was incorrectly entered into one of the databases (age of infant should be 38 weeks instead of 29 weeks). The age of the infant was changed in the article. This change did not alter the data interpretation or conclusions of the study.

