# Supplementary material for: Premature infants display discriminable behavioral, physiological, and brain responses to noxious and nonnoxious stimuli
Source: Cereb Cortex. 2021 Dec 28;32(17):3799–815. doi: 10.1093/cercor/bhab449 (PMC9433423; doi:10.1093/cercor/bhab449)
Supplement: Supplementary_Materials_for_Premature_infants_display_discriminable_responses_clean_20-01-2022_bhab449.pdf [file supplementary_materials_for_premature_infants_display_discriminable_responses_clean_20-01-2022_bhab449.pdf]

Supplementary materials for

Premature infants display discriminable behavioural, physiological and brain responses to noxious and non-noxious stimuli

Marianne van der Vaart[1], Caroline Hartley[1], Luke Baxter[1], Gabriela Schmidt Mellado[1], Foteini Andritsou[1], Maria M. Cobo[1,4], Ria Evans Fry[1], Eleri Adams[2], Sean Fitzgibbon[3]* and Rebeccah Slater[1]*

*These authors contributed equally to this work

Affiliations:

[1]Department of Paediatrics, University of Oxford, Oxford OX3 9DU, UK

[2]Newborn Care Unit, John Radcliffe Hospital, Oxford University Hospitals NHS Foundation Trust, Oxford, OX3 9DU, UK

[3]Wellcome Centre for Integrative Neuroimaging, FMRIB, Nuffield Department of Clinical Neurosciences, University of Oxford, UK

[4]Universidad San Francisco de Quito USFQ, Colegio de Ciencias Biologicas y Ambientales, Quito, Ecuador

Corresponding author: Prof. Rebeccah Slater

Telephone: +44 1865 234 229

Email: rebeccah.slater@paediatrics.ox.ac.uk

Address: Department of Paediatrics, University of Oxford, Level 2, Children's Hospital, John Radcliffe, Headington, Oxford, OX3 9DU

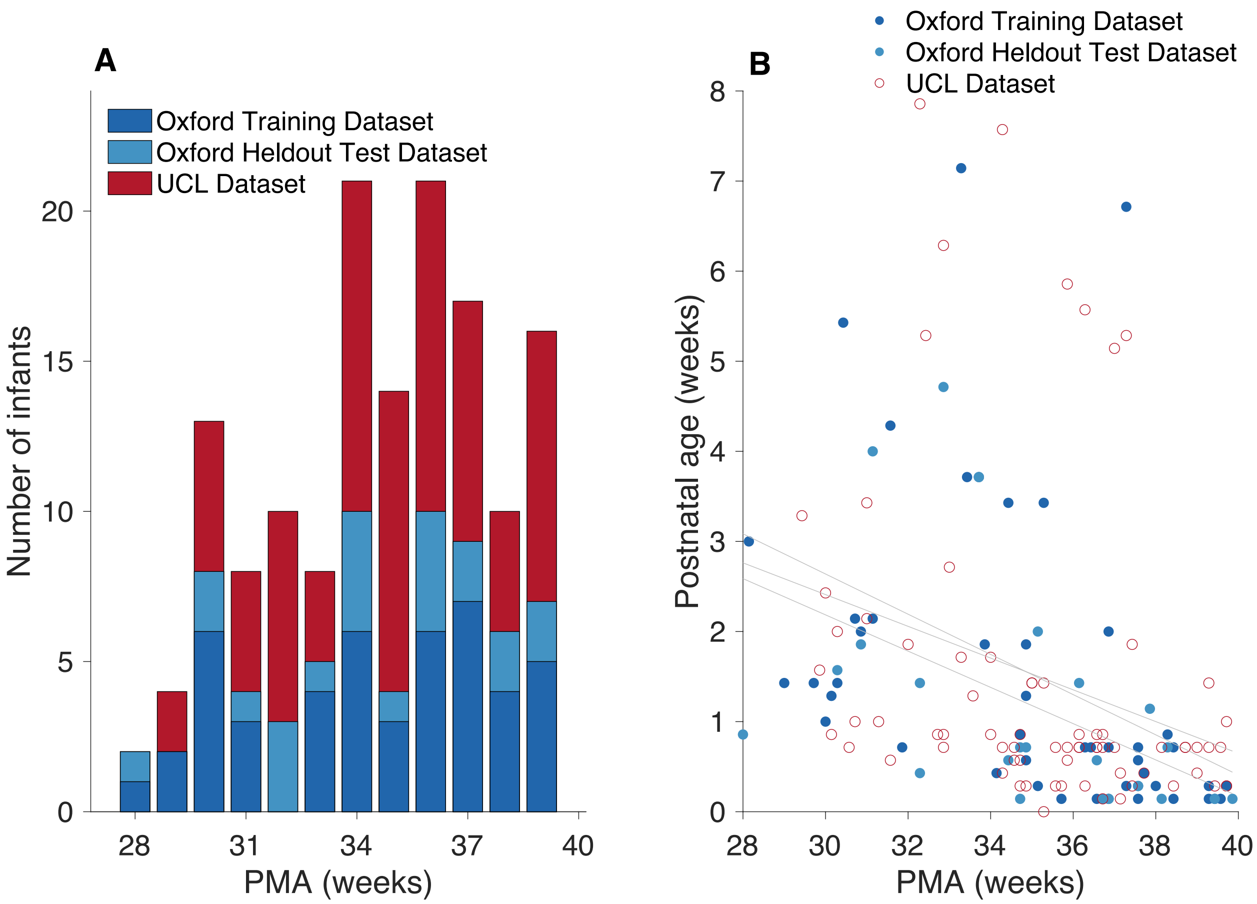

Figure S1. Age distribution in the Oxford and UCL Datasets. A) Distribution of postmenstrual age (PMA) in the Oxford Training Dataset, Oxford Held-out Test Dataset and UCL Dataset. B) Relationship between PMA and postnatal age. Grey lines are the lines of best fit in the three datasets.

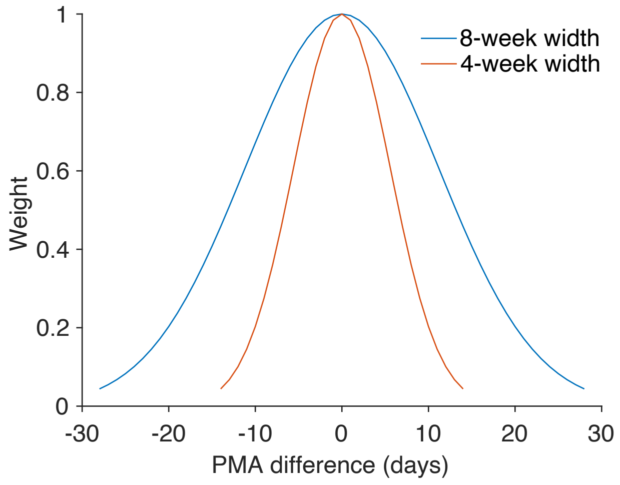

Figure S2. Gaussian windows used to assign weights for the calculation of age-weighted averages before the principal component analysis (8-week width) and the age trajectories presented in Figures 4-7 (4-week width).

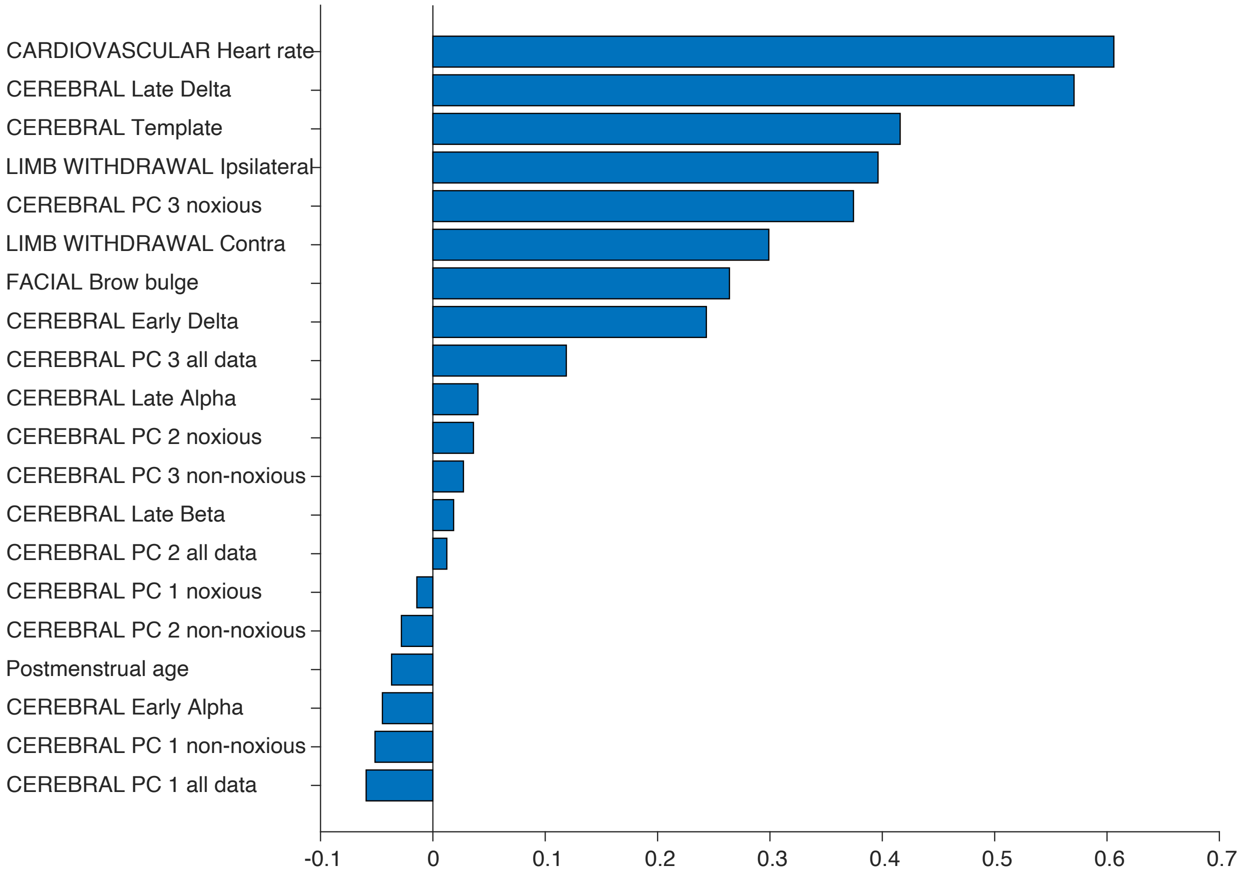

Figure S3. Permutation feature importance estimated in the out-of-bag samples in the training data. Abbreviations: PC = principal component.

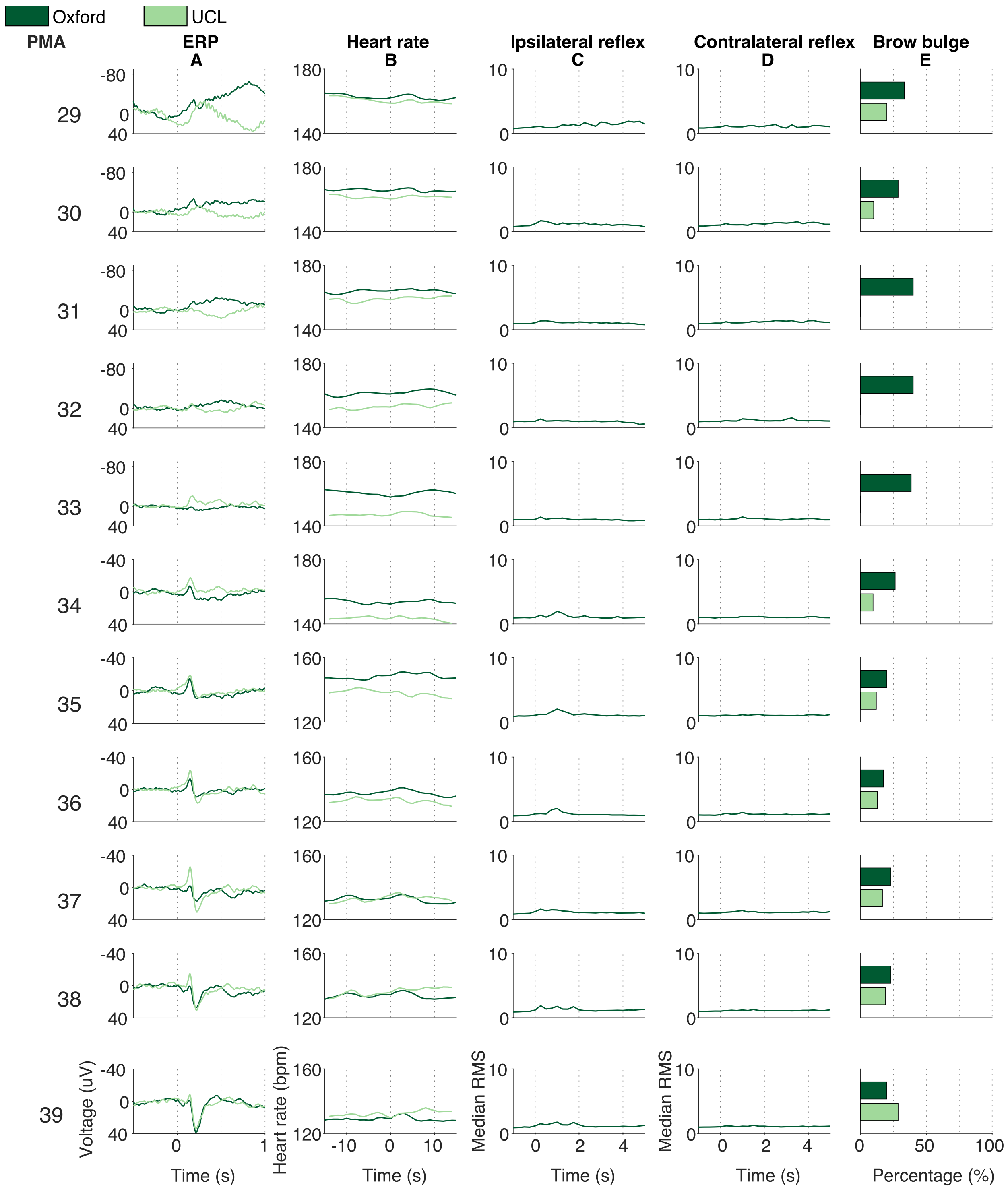

Figure S4. ERP, heart rate, ipsilateral reflex, contralateral reflex and brow bulge responses to the non-noxious control procedure in neonates from 28-40 postmenstrual weeks, split by postmenstrual week. For the continuous variables, each trace is an age-weighted average (ERP,

heart rate) or median (reflex) in 4-week sliding windows around the centre PMA. For the brow bulge responses, bars demonstrate the percentage of infants that displayed a brow bulge response in a group of infants with a PMA that falls within 1.5 weeks relative to the centre PMA. Oxford data contains the Oxford training set and the Oxford Held-out Test Dataset. EMG is not available in the UCL Dataset. Column A) ERP. Column B) Heart rate. Columns C-D) Ipsilateral and contralateral reflex responses. Column E) Proportion of infants displaying a brow bulge response. Abbreviations: ERP = event-related potential; PMA = postmenstrual age.

Table S1. Regression coefficients and statistics for associations between (left) PMA and noxious-response metrics corrected for PNA, and (right) PNA and noxious-response metrics corrected for PMA. Noxious-response metrics are sorted by p-value for the PMA covariate. Results are consistent with the analyses in the main text. P-values are uncorrected for multiple testing due to the exploratory nature of the analyses. Abbreviations: PC = principal component.

	**PMA**			**PNA**		
	**Beta**	**t-stat**	**p**	**Beta**	**t-stat**	**p**
PC 1	-0.018	-5.41	0.0001	-0.007	-1.24	0.21
PC 2	-0.022	-6.14	0.0001	0.006	0.90	0.36
Heart rate	1.63	6.42	0.0001	-0.29	-0.66	0.51
PC 3	0.010	2.89	0.005	-0.012	-1.85	0.070
Early Alpha	-0.48	-2.44	0.018	0.37	0.93	0.36
Late Delta	0.55	2.15	0.033	1.18	2.33	0.023
Early Delta	-0.40	-1.92	0.058	0.61	1.46	0.15
Late Beta	0.40	1.67	0.10	1.25	2.66	0.012
Ipsilateral reflex	-0.22	-1.03	0.31	-0.64	-1.36	0.17
Contralateral reflex	0.17	0.64	0.53	-0.21	-0.39	0.69
Late Alpha	-0.12	-0.61	0.55	1.14	2.81	0.010
